# Regional health differences – developing a socioeconomic deprivation index for Germany

**DOI:** 10.17886/RKI-GBE-2017-048.2

**Published:** 2017-06-14

**Authors:** Lars Eric Kroll, Maria Schumann, Jens Hoebel, Thomas Lampert

**Affiliations:** Robert Koch Institute, Department for Epidemiology and Health Monitoring, Berlin, Germany

**Keywords:** SOCIAL DEPRIVATION, HEALTH INEQUALITIES, GERMAN INDEX OF SOCIOECONOMIC DEPRIVATION (GISD), FACTOR ANALYSIS

## Abstract

For Germany, regional differences for various health indicators, which are also associated with socioeconomic factors, have been documented. This article aims to develop a regional socioeconomic deprivation index for Germany that (1) can be used to analyse regional socioeconomic inequalities in health and (2) provides a basis for explaining regional health differences in Germany.

The core data stem from the INKAR (indicators and maps on spatial and urban development in Germany and Europe) database compiled by Germany’s Federal Institute for Research on Building, Urban Affairs and Spatial Development. Factor analysis is used for indexing and the weighting of indicators for the three dimensions of education, occupation and income. The German Index of Socioeconomic Deprivation (GISD) is generated at the levels of associations of municipalities, administrative districts and administrative regions for the years 1998, 2003, 2008 and 2012. Aggregate data and individual data from the German Health Update 2014/2015-EHIS (GEDA 2014/2015-EHIS) study are used to analyse associations between the index and selected health indicators. For around two thirds of the causes of death, the German Index of Socioeconomic Deprivation reveals significant socioeconomic inequalities at the level of Germany’s administrative regions. At district level, life expectancy in the bottom fifth of districts presenting the highest levels of deprivation is, depending on the observation period, 1.3 years lower for women and 2.6 years lower for men in comparison to the upper fifth of districts presenting the lowest levels of deprivation. The index can explain 45.5% and 62.2% of regional differences in life expectancy for women and men, respectively. Moreover, the population in regions characterised by high levels of deprivation has significantly higher rates of smokers, engages less frequently in leisure-time physical activities and is more often obese.

The German Index of Socioeconomic Deprivation illustrates regional socioeconomic differences at different spatial levels and contributes to explaining regional health differences. This index is intended for use in research as well as by federal and federal state health reporting systems and should enable access to new sources of data for investigating the links between social inequalities and health in Germany.

## 1. Introduction

To provide the most comprehensive and precise picture of health in Germany, Federal Health Reporting (GBE) uses numerous sources of data. In addition to health surveys carried out by the Robert Koch Institute, as well as sociological and epidemiological studies, these include official statistics and process-produced data from social insurers [1]. Robust conclusions depend on representative, valid and reliably processed information. Moreover, to reflect trends, this information should be collected continuously. Regional and social health disparities are a focus of health reporting [1].

This approach of the GBE fulfils the requirements of the World Health Organization (WHO), which regards continuous monitoring of the scale of health inequalities and providing evidence of measures to reduce such inequalites as a central task for public health [2]. Numerous international surveys have demonstrated that access to healthcare, disease risks and also life expectancy are distributed unequally in most countries [3]. Socially disadvantaged individuals tend to view their health as being poorer than those who are better off, they do also display riskier behaviour with regard to their health and face higher disease burdens and mortality. These inequalities in health chances are also present in Germany [4-6]. Moreover, regarding various health indicators, pronounced regional health differences exist in Germany that are also related to social characteristics of particular regions [5, 7, 8].

Frequently, the description of social inequality is based on measures of socioeconomic status (SES) for individuals or households. The underlying assumption here is that socioeconomic status is, in most cases, related to particular social advantages and disadvantages defined as individual access to scarce resources highly valued in society, such as money, wealth, power, social prestige, education and knowledge [9]. Education, occupational status and income are seen to constitute the central defining factors for socioeconomic status and the core dimensions of social inequality [10, 11]. Social and health surveys therefore collect this information to define the socioeconomic status of respondents. This is done both by using the single indicators (education, occupation and income) separately and by using composite status indices [12-14].

Numerous data sources for health reporting, however, provide hardly any information on the individual socioeconomic situation of the people included. This makes analysing social inequalities in health very difficult. In Germany, this particularly applies to the data concerning life expectancy and causes of death, cancer registries, statistics regarding absences from work, as well as diagnosis data from outpatient and inpatient care. Due to strict data protection regulations, some of these data sources often only provide regionally aggregated data. In order to be able to analyse social inequalities in health, such data are then often related to regional socioeconomic indicators. Such indicators can pinpoint a region’s social conditions. Possible indicators include the at-risk-of-poverty rate [15], unemployment rates, household income per capita [8] or multidimensional indices [17, 18].

Multidimensional indices at the regional level offer the benefit of highlighting not merely individual aspects, but the overall set of socioeconomic advantages and disadvantages of a region. Within this context, international research often uses the term social or socioeconomic deprivation. Applied to individuals, the concept describes a relative lack of material resources; compared to others, the person in question has so few resources at their disposal that their participation in social activities is potentially limited [19, 20]. When applied to regions, however, the term deprivation highlights the fact that socio-spatial resources and burdens can also impact social participation. Measures of regional deprivation have been used in England since the 1980s to make compare regions regarding their associated need for healthcare [20]. Most indices thereby build on the concepts developed by Townsend [19], Carstairs [21] and Jarman [22]. Beyond socioeconomic indicators, more recent indices on so called “multiple deprivation” [23] consider further indicators, such as the life expectancy of the population. Whilst multiple deprivation approaches are better at explaining regional differences in care needs, they are of limited value to epidemiological research because, at a conceptual level, they do not clearly discriminate between health determinants and the consequences of diseases [20].

This article aims to develop a regional deprivation index for Germany that is capable of demonstrating regional socioeconomic inequalities. The index uses internationally established indicators and is based on the concept of socioeconomic status as it is used in social epidemiology to describe the social situation of individuals and households [14]. The following sections describe the key elements of the socioeconomic deprivation index and provide an analysis of statistical associations between the index and several health indicators at different regional levels. Finally, we discuss the index’s potential and limitations with regard to research and health reporting.

## 2. Data and method

### 2.1 Data sources

The data source for regional socioeconomic information is the INKAR (indicators and maps on spatial and urban development) database compiled by Germany’s Federal Institute for Research on Building, Urban Affairs and Spatial Development (BBSR) [24]. INKAR is an interactive online database containing regional statistics for Germany and Europe. Indicators are available for different regional levels. This makes comparisons between European regions, German federal states, districts, central areas and associations of municipalities possible. Most statistics date back to 1995 and analyse a consistent territory (as of 31 December 2014). Currently, the database contains around 600 indicators providing information on population, labour market, income and earnings, housing, education, social and medical care, transport and accessibility, land use and the environment, as well as public finances and budgets.

Regional health information for initial relation analysis was also acquired from the INKAR database, as well as from the statistical office of the European Union (Eurostat). Regional data are available for different spatial levels (Table 1). The INKAR database provides data on life expectancy at birth for the 402 rural districts and towns not attached to an administrative district (termed districts in the following sections). Eurostat provides age-standardised information on mortality differentiated according to ICD-10 chapters (International Statistical Classification of Diseases and Related Health Problems, tenth revision) for the European administrative divisions NUTS-2 (Nomenclature of territorial units for statistics). According to official European statistics, for Germany this administrative level comprises 39 administrative or statistical regions.

Regionalised health information based on individual data was taken from the 2014/2015 German Health Update study (GEDA 2014/2015-EHIS). GEDA is part of health monitoring at the Robert Koch Institute (RKI) and has been regularly conducted as a cross-sectional health survey of adults (aged over 18) since 2009 [25]. The sample was conceived as a two-step cluster sample. In a first step, 301 municipalities and associations of municipalities stratified by federal state and BIK classification were selected randomly out of all the municipalities in Germany [26]. The probability of a municipality being drawn was thereby proportional to the size of its population [27]. In the selected municipalities, random samples from the residents’ registration office were taken. The response rate was 27.7%. The statistical analysis was carried out using weighting factors that correct deviations of the sample from the German population (as of 31 December 2014) with regard to gender, age, district type and education. In total, data from 24,016 women and men aged over 18 were used. A detailed description of the methodology applied in the GEDA 2014/2015-EHIS survey can be found in the article German Health Update – New data for Germany and Europe in issue 1/2017 of the Journal of Health Monitoring.

### 2.2 Indicators to develop the socioeconomic deprivation index

To select suitable indicators for the German Index of Socioeconomic Deprivation (GISD), we conducted a comprehensive research of literature in the Pubmed and Google Scholar databases, which yielded 372 international articles on regional deprivation. After excluding double and irrelevant hits, 49 articles to extract indicators remained. To be shortlisted, an indicator had to be closely connected to one of the three central dimensions of socioeconomic status (education, occupation and income) [10]. In a final step, we verified the availability of the corresponding indicators in the INKAR database and selected indicators that are available at the district or associations of municipalities level for the period from 1998 to 2012. Unfortunately, regarding the dimensions of education and occupation, only very few indicators fulfilled these criteria. Slightly more data are available for the dimension of income.

Unemployment rates in a region, the average gross wage of employees and the employment rate were selected as indicators for the dimension of occupation. Gross wage is used as an indicator for the mean occupational status of employees in a region as it is the best indicator available. The dimension of education used the share of employees with a university degree and the share of those who leave school without a certificate. The indicators for monthly mean net household income, debtor quotas and tax revenue were used for the dimension of income (Table 2). For those indicators for which no complete data sets for the years 1998 to 2012 exist, missing values at the district level were estimated based on regression analysis (linear random intercept model time series). For the five indicators that were only available at the district level, values for associations of municipalities were estimated by regression analysis based on other available indicators (Table 2). This means that at the level of associations of municipalities the index is associated with greater uncertainties than at the district level. Furthermore, the index for 1998 is less precise than for the following years as for this particular year data were unavailable for several indicators.

### 2.3 Index development

Analogous to the approach adopted in international literature, during index development, a factor analysis was performed to weight the indicators for the three dimensions of socioeconomic deprivation [28-31]. Rotated factor loadings were used and a single factor solution indicated for each dimension. The three generated factors were given equal weighting in the resulting index, i.e. each contributing one third (Table 3). For the dimension of education, there were only two indicators, which meant a factor analysis was not applicable. Because employees represent a notably larger proportion of the population, the indicator education status of employees was given twice the weight of the indicator proportion of people who leave school without a certificate based on school statistics. This was done in consideration of the fact that the ratio of employees (education status of employees) compared to households of adults with children (school leavers without certificates) is roughly two to one. In the absence of conclusive indicators for education at the regional level, values are approximate estimates.

The index was standardised for each survey year and each spatial level (associations of municipalities, districts, administrative regions [NUTS-2], spatial planning regions), which means that the regional socioeconomic deprivation index can vary between 3 (lowest degree of deprivation/highest socioeconomic status) and 21 (highest degree of deprivation/lowest socioeconomic status). Standardisation aimed to ensure the comparability of the variation range with the composite index of individual socioeconomic status developed for the health surveys conducted by the Robert Koch Institute. Moreover, the units of the mentioned spatial levels, i.e. the corresponding regions, were weighted according to the population for further analysis of the distribution of index values for each year and categorised in two ways. First, they were divided into groups of 20% (quintiles, fifths) weighted by their population. These quintiles were then used to differentiate between regions with low (lowest quintile), medium (middle three quintiles) and high (highest quintile) levels of socioeconomic deprivation. The variation range of 3 to 21 points and category development was guided by the development of individual socioeconomic status in population-wide epidemiological surveys in the context of health monitoring conducted by the Robert Koch-Institute [14].

### 2.4 Analysis strategy

The following section presents the regional distribution of the index and results on associations between regional socioeconomic deprivation and average life expectancy as well as the individual health indicators smoking, leisure-time physical inactivity and obesity. Moreover, the associations between regional socioeconomic de-privation and individual socioeconomic status are highlighted.

The German Index of Socioeconomic Deprivation was linked to district identifiers. As a measure to quantify the association between the index and health indicators, the Relative Index of Inequality (RII) was calculated [32]. This regression-based measure takes into account the entire distribution of a socioeconomic variable. In the following, the RII can be interpreted as the estimated rate ratio between people living in regions with the highest and those living in regions with the lowest level of socioeconomic deprivation. A value of 1 translates as no regional socioeconomic inequalities; values greater than 1 indicate an increased rate in deprived regions, whereas values between 0 and 1 indicate a lower rate in deprived regions. In contrast, the Slope Index of Inequality (SII) was used to analyse associations between regional socioeconomic deprivation and life expectancy. Analogous to the Relative Index of Inequality, it describes the absolute difference in life expectancy [32]. The SII was required because no age-standardised mortality figures to calculate the RII were available at the district level. All analysis was conducted using the Stata SE 14.1 statistical package.

## 3. Results

**Figure 1:**
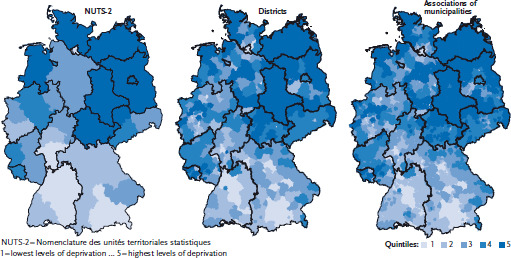
Regional levels of socioeconomic deprivation (in quintiles) by spatial levels in Germany 2012 Data sources: INKAR, own calculations

Figure 1 shows the distribution of the German Index of Socioeconomic Deprivation at the level of associations of municipalities, districts and administrative regions or statistical regions according to the official European statistics (NUTS-2) for 2012. Overall, the figures show that levels of socioeconomic deprivation are spread unevenly between the West German and the East German federal states (also known as the new federal states). Many associations of municipalities presenting high values for socioeconomic deprivation are located in the new federal states; however, further concentrations can also be found in the Saarland, North Rhine-Westphalia and rural areas of Lower Saxony. Areas where the levels of socioeconomic deprivation tend to be low are found mainly in Bavaria, Baden-Württemberg, Hesse and parts of North Rhine-Westphalia, such as in Düsseldorf and the Cologne/Bonn region.

**Figure 2:**
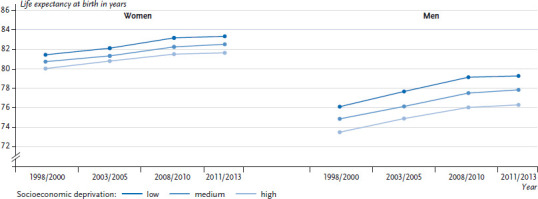
Regional levels of socioeconomic deprivation (in categories at the district level) and life expectancy Data sources: indicators and maps on spatial and urban development (INKAR); own calculations

Figure 2 shows the differences in life expectancy at the level of districts for the years 1998/2000 through to 2011/2013. Socioeconomic deprivation is classified into the three categories low, medium and high. For the observation period, men from districts with low levels of deprivation had a mean life expectancy that was 2.9 years higher than for men from the most deprived districts (SII=3.44). For women, the corresponding mean difference was 1.5 years (SII=1.86). Over the entire observation period, the regional socioeconomic inequalities in mean life expectancy measured using the SII increased significantly by 27.7% for women and 20.2% for men. Expressed in years, the difference in life expectancy between districts with high and low levels of deprivation increased from 1.4 to 1.7 years for women and 2.6 to 3.0 years for men during the period of observation. The German Index of Socioeconomic Deprivation can statistically explain 45.5% (adjusted R^2^) of regional differences in life expectancy for women and 62.2% for men.

Table 4 shows the causes of death (ICD-10 disease chapters) where regional socioeconomic inequalities in mortality at the level of administrative and statistical regions were particularly large between 2008 and 2010. The Relative Index of Inequality reveals significant socio-spatial disparities with regard to total mortality and diseases of the circulatory system (I00–I99), for neoplasms (C00–D48), diseases of the respiratory system (J00–J99) and diseases of the digestive system (K00–K93, only for men) and, therefore, for 80.7% of all deaths in the period considered.

**Figure 3:**
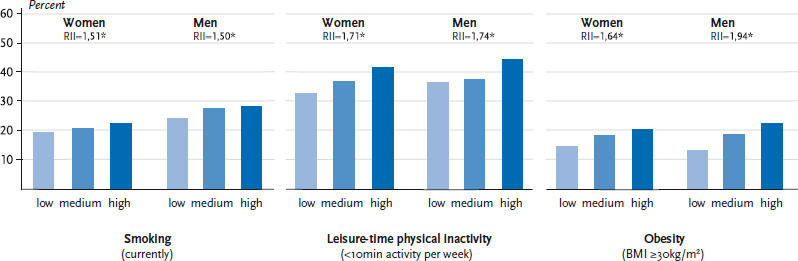
Regional levels of socioeconomic deprivation (in categories at the level of associations of municipalities) and behaviour-related individual risk factors Data sources: GEDA 2014/15-EHIS; INKAR; own calculations

Beyond the described statistical associations at the regional level, data from the Robert Koch Institute’s GEDA 2014/2015-EHIS survey can provide a link between regional values for socioeconomic deprivation and the individual health of respondents. In the 255 associations of municipalities in which GEDA respondents lived, the three health risks smoking (answering the question ‘Do you smoke?’ with ‘yes, daily’ or ‘yes, occasionally’), leisure-time physical inactivity (<10 minutes of leisure-time physical activity per week) and obesity (body mass index ≥30 kg/m^2^) are significantly more prevalent in associations of municipalities with higher levels of socioeconomic deprivation than in those with comparatively low levels of deprivation (Figure 3). With the exception of obesity, the link with levels of socioeconomic deprivation is similarly strong for women and men. When comparing associations of municipalities with the highest levels of socioeconomic deprivation to those with the lowest, the Relative Index of Inequality is 1.5 to 1.7. For male obesity, it is 1.9.

Moreover, GEDA reveals the varying statistical importance of individual socioeconomic status and regional socioeconomic deprivation for the spread of health risks. Table 5 shows the results from four gradually calculated regression models for the considered health risks. In a first step, the general regional variation of health risks at the level of associations of municipalities (M0) is considered. In the following steps, the links with regional socioeconomic deprivation (M1), individual socioeconomic status (SES) (M2), as well as the interaction between both of these factors (M3) are taken into account. When interpreting results, SES index and German Index of Socioeconomic Deprivation scores must be interpreted inversely. High SES index scores point to a better individual socioeconomic situation, high scores in the German Index of Socioeconomic Deprivation point to a worse regional socioeconomic situation. The results therefore show that both regional levels of socioeconomic deprivation and individual socioeconomic status have significant and independent links to health risks. The higher an individual’s socioeconomic status is, the lower the prevalence of smoking, leisure-time physical inactivity and obesity. Yet, in regions characterised by high levels of socioeconomic deprivation, these risk factors are generally more prevalent, independent of individual socioeconomic status. Moreover, results from the interaction model (M3) indicate that a person’s individual socioeconomic status has no significant impact on this link between regional socioeconomic deprivation and health risks. One exception is smoking among women, where a marginally significant interaction effect (p<0.10) was observed.

## 4. Discussion

This study introduces a new index for regional socioeconomic deprivation in Germany. The German Index of Socioeconomic Deprivation (GISD) operationalises regional deprivation multi-dimensionally at the population level based on the three equally weighted dimensions of education, occupation and income. Initially generated for the years 1998, 2003, 2008 and 2012, the index will be updated regularly every few years. Initial association analysis revealed a certain degree of statistical link between regional differences in life expectancy, major causes of death and behavioural health risks with levels of regional socioeconomic deprivation. Further analysis suggested that, to a certain extent, individual socioeconomic status can mediate the relation between regional deprivation and behavioural health risks: Statistically controlling for individual socioeconomic status substantially reduces the effect of regional deprivation, but in most cases does not totally explain its effect. Overall, the results indicate that individual socioeconomic status is not an effect modifier because there is no significant difference in the statistical link between regional deprivation and behavioural health risks among women and men with a low socioeconomic status compared to those with a higher status.

The findings are in line with German and international literature. Health in regions with higher levels of socioeconomic deprivation tends to be worse, as does behaviour with regard to health [34-40]. Similar studies in countries such as England and New Zealand have also shown lower life expectancy at birth and the reduction of later life expectancy with increasing levels of socioeconomic deprivation in specific regions [34-36]. Corresponding links regarding regional unemployment rates, average income and at-risk-of-poverty rates have also been documented in Germany [5, 6, 16]. Moreover, a link with regional deprivation markers and mortality was shown: mortality rates in deprived regions are higher than average [37, 38]. In terms of individual health outcomes, in Germany increasing degrees of deprivation translate into higher rates of obesity [39], smoking and physical inactivity [40].

In terms of methodology, the utilized approach is in line with the discussion taking place internationally. For example, the New Zealand Deprivation Index (NZDep) [28, 29], the Deprivation Index for Quebec and Canada (INSPQI) [41-43], the French small-area index of socioeconomic deprivation [30], the Deprivation index for small areas in Spain [44] and the Danish Deprivation Index (DANDEX) [31] also use factor analysis to weight indicators within the different dimensions of regional socioeconomic deprivation.

The approach has certain advantages but also limitations. Many deprivation indices that build on the work by Townsend [19], Carstairs [21] and Jarman [22] are based on census data. For Germany, however, census data are only available in irregular intervals. Process-produced data were therefore mainly used to be able to regularly update the index. However, this means that, overall, there are only scant conclusive indicators, in particular at the level of associations of municipalities. Moreover, some standard of life indicators, such as passenger car density, were not used to increase the comparability of index values between urban and rural regions [45-48].

Applying the index at the level of associations of municipalities increases the socioeconomic homogeneity of units compared to the district level and decreases the risk of false conclusions due to the effect of administrative boundaries (modifiable areal unit problem) [49]. Factor analysis allows a better use of the available information than if it were weighted equally [19, 21] and the approach is less prone to systematic bias than subjective weighting by experts as is occasionally applied in some countries [22, 50]. However, compared to individual socioeconomic status, the applicability of deprivation indices is limited. They can be used to identify socioeconomically deprived regions, but allow no conclusions on individual socioeconomic status [18, 19, 51, 52] or the extent of health inequalities in a determined region [53].

In our view, the generated deprivation index is a useful additional tool for research and health reporting. Limiting the index to socioeconomic indicators ensures a clear interpretation of statistical associations. The index thereby complements data on individual socioeconomic status and allows for conclusions on independent explanations of regional socioeconomic deprivation and interactions with individual socioeconomic status. Where an individual operationalisation of socioeconomic status is not possible (for example, in the data of the cause of death statistics in Germany), the index, at least to a certain degree, reveals the extent of health inequalities and provides additional reasons to collect individual data [41]. Moreover, the results can be used as a basis for health policy initiatives and for the development of health promotion and prevention strategies to achieve substantial change in regions with high levels of socioeconomic deprivation. As regional analyses have the potential to promote a targeted allocation of financial resources, they also have the potential to promote health equality [28, 31].

The GISD is provided free to use for research and health reporting at the data archive datorium of the German GESIS [54].

## Key statements

- The German Index of Socioeconomic Deprivation reflects regional socioeconomic differences at different spatial levels.
- The index implements eight indicators from the three core dimensions of social inequality – education, occupation and income.
- In parts of the East German federal states, but also in the Saarland, North Rhine-Westphalia and Lower Saxony, levels of socioeconomic deprivation are higher.
- Life expectancy is lower, mortality is higher and the health risks are greater in regions with higher levels of socioeconomic deprivation.
- The German Index of Socioeconomic Deprivation is available for research and health reporting on different spatial levels and for various years.
- The index provides the field of health reporting with new data sources to analyse health inequilities.

## Figures and Tables

**Table 1:** Administrative levels in Germany Source: BBSR [24]

Level	Number of areas	Average population	Range of population figures	
			Minimum	Maximum
Associations of municipalities (GVB)	4,504	17,878	338	3,375,222
Districts and towns not attached to an administrative district (districts)	402	200,308	34,064	3,375,222
Spatial planning regions (ROR)	96	838,789	203,544	3,375,222
NUTS-2	39	2,064,711	518,289	5,081,061

NUTS-2= Nomenclature of territorial units for statistics, EU statistical regions, level 2, basic regions, corresponds to administrative districts or statistical regions of federal states. Territorial units and population as of 31 December 2012

**Table 2:** Indicators of socioeconomic deprivation Source: INKAR [24]

Category	Indicator	Statistical source	Availability
Unemployed	Proportion of people unemployed as share of working age residents in %	Statistics of the Federal Employment Agency	Associations of municipalities for the years 1998, 2003, 2008, 2012
Employees at place of residence with university degree	Proportion of employees with social insurance at place of residence with university degree as share of employees with social insurance at place of residence in %	Statistics of the Federal Employment Agency	Districts for the years 1999, 2003, 2008, 2012
Employment quota	Proportion of employees with social insurance at place of residence per 100 working age inhabitants	Statistics of the Federal Employment Agency	Associations of municipalities for the years 2003, 2008, 2012, districts for 1998
Gross wages and salaries	Gross wage and salary in EUR per employee	Official federal and federal state employment statistics	Districts for the years 2000, 2003, 2008, 2012
Net household income	Average household income in EUR per inhabitant	National Accounts Working Group (Arbeitskreis Volkswirtschaftliche Gesamtrechnung der Länder)	Districts for the years 2000, 2003, 2008, 2012
School leavers without certificate	Proportion of school leavers without school-leaving certificate out of all school leavers in %	Statistics on the schools of general education	Districts for the years 1998, 2003, 2008, 2012
Debtor quota	Private debtors per 100 inhabitants aged 18 and above	Statistics from creditreform e.V. associations	Districts for the years 2004, 2008, 2012
Tax revenue	Tax revenue in EUR per inhabitant	Comparison of federal and federal state taxation on real estate and working assets	Associations of municipalities 2003, 2008, 2012, districts for 1998

**Table 3:** Weighting of indicators for socioeconomic deprivation in the three subdimensions of the German Index of Socioeconomic Deprivation Data sources: INKAR, own calculations

Dimension (Proportion of GISD)	Indicator (z-standardised)	Factor loading	Correlation of indicators with dimension (Pearson)
Education (33.3%)	School leavers without certificate	-0.33	0.76
	Employed at place of residence with university degree	+0.66	-0.74
Occupation (33.3%)	Unemployed	-0.61	0.89
	Gross income and wage	+0.27	-0.63
	Employment quota	+0.50	-0.55
Income (33.3%)	Debtor quota	-0.41	0.70
	Net household income	+0.52	-0.88
	Tax revenue	+0.39	-0.55

**Table 4:** Socioeconomic deprivation (in categories at the level of administrative and statistical regions) and deaths (2008-2010) by cause of death Data sources: Statistical Office of the European Union (Eurostat) [33]; own calculations

Cause of death according to the main groups listed in the ICD-10 and arranged based on the proportion of age-standardised deaths		Share in causes of death	Standardised mortality rate per 100,000 residents	By socioeconomic deprivation (GISD)		Relative Index of Inequality (RII) by gender		
Code	Description	Total	Total	Low	High	Total	Women	Men
A-R, V-Y	Total mortality excluding chapters S, T and Z	100.0%	1063.8	977.9	1135.2	1.19	1.15	1.24
I00-I99	Diseases of the circulatory system	42.2%	449.2	396.8	507.3	1.26	1.24	1.29
C00-D48	Neoplasms	26.0%	276.3	261.0	285.8	1.15	1.08	1.24
J00-J99	Diseases of the respiratory system	7.6%	80.3	67.6	81.6	1.22	1.19	1.29
K00-K93	Diseases of the digestive system	4.9%	52.4	50.2	55.4	-	-	1.17
V01-Y98	External causes of morbidity and mortality	3.6%	38.8	43.3	41.2	-	-	-
E00-E90	Endocrine, nutritional and metabolic diseases	3.3%	35.6	35.0	41.1	-	-	-
F00-F99	Mental and behavioural disorders	2.7%	28.3	27.2	26.5	-	0.77	-
R00-R99	Symptoms, signs and abnormal clinical and laboratory findings, not elsewhere classified	2.4%	25.9	20.9	22.7	2.46	2.42	2.54
G00-G95	Other diseases of the nervous system	2.4%	25.9	27.0	24.6	-	-	-
N00-N99	Diseases of the genitourinary system	2.3%	24.4	21.3	27.2	1.27	1.31	1.24
A00-B99	Certain infectious and parasitic diseases	1.7%	18.3	17.5	13.3	-	-	-
M00-M99	Diseases of the musculoskeletal system and connective tissue	0.3%	3.2	4.4	2.8	-	0.50	-
D50-D89	Diseases of the blood and blood-forming organs and certain disorders involving the immune mechanism	0.3%	3.2	3.4	3.5	-	-	-
L00-L99	Diseases of the skin and subcutaneous tissue	0.1%	1.1	1.2	1.0	-	-	-
Q00-Q99	Congenital malformations, deformations and chromosomal abnormalities	0.1%	0.9	1.0	1.1	-	-	-
O00-O99	Pregnancy, childbirth and the puerperium	0.0%	0.1	0.1	0.1	-	-	-
P00-P96	Certain conditions originating in the perinatal period	0.0%	0.0	0.0	0.0	-	-	-

Legend: GISD=German Index of Socioeconomic Deprivation; ICD-10=International Statistical Classification of Diseases and Related Health Problems, 10th Revision; RII=Relative Index of Inequality; “–”=nonsignificant results Eurostat statistics do not record data for ICD-10 codes S00-T98 and Z00-99. Total excluding codes O00-O99. Standardised mortality rates per 100,000 residents: age-standardised deaths per 100,000 residents (revision of the European Standard Population 2013). Standardised mortality rates by socioeconomic deprivation: mortality rate at NUTS-2 level (nomenclature of territorial units for statistics) differentiated by levels of socioeconomic deprivation (in categories). Relative Index of Inequality according to GISD=Relative Index of Inequality of mortality rates by levels of socioeconomic deprivation.

**Table 5:** Link between individual and regional socioeconomic deprivation and behaviour-related risk factors; results from multilevel logistic regression modelling Data sources: GEDA 2014/15-EHIS; own calculations

	Smoking		Physical inactivity		Obesity	
	Women	Men	Women	Men	Women	Men
**M0: Basic model**						
MOR(GVB)	1.18^*^	1.13^*^	1.19^*^	1.29^*^	1.29^*^	1.31^*^
**M1: Deprivation**						
RII(GISD)	1.51^*^	1.50^*^	1.71^*^	1.74^*^	1.64^*^	1.94^*^
MOR(GVB)	1.12^*^	1.03	1.10^*^	1.22^*^	1.23^*^	1.22^*^
**M2: Deprivation and SES**						
RII(GISD)	1.24^*^	1.25^*^	1.38^*^	1.32^*^	1.38*	1.66^*^
RII(SES)	0.25^*^	0.36^*^	0.21^*^	0.19*	0.27^*^	0.41^*^
MOR(GVB)	1.18^*^	1.07	1.00	1.20*	1.18^*^	1.19^*^
**M3: Deprivation and SES interaction**						
RII(GISD)	1.60^*^	1.42^*^	1.58^*^	1.18	1.21	1.31
RII(SES)	0.33^*^	0.41^*^	0.24^*^	0.17^*^	0.23^*^	0.32^*^
RII(GISD)*RII(SES)	0.61°	0.78	0.77	1.23	1.31	1.58
MOR(GVB)	1.17^*^	1.06°	1.00	1.20^*^	1.18^*^	1.19^*^

Controlled for age (metric and squared) levels: associations of municipalities and individual. Significant impact of variables and/or variation at spatial level *= p<0.05 or marginally significant °=p<0.10. SES=individual socioeconomic status; GISD=German Index of Socioeconomic Deprivation; RII=Relative Index of Inequality; MOR=Median Odds Ratio for levels of associations of municipalities.
