# Corrigendum: Regional health differences – developing a socioeconomic deprivation index for Germany

**DOI:** 10.25646/6793

**Published:** 2018-10-18

**Authors:** Lars Eric Kroll, Maria Schumann, Jens Hoebel, Thomas Lampert

Kroll LE, Schumann M, Hoebel J et al. (2017)

Journal of Health Monitoring 2(2): 98-114.

DOI 10.17886/RKI-GBE-2017-048

In the original article, in Table 4 ‘Socioeco-nomic deprivation (in categories at the level of administrative and statistical regions) and deaths (2008-2010) by cause of death’ some of the values for standardised mortality rates by socioeconomic deprivation (GISD) were switched in the columns ‘Low’ and ‘High’.

The corrected version of the article is available at DOI 10.17886/RKI-GBE-2017-048.2

